# Promoting lab culture to enhance academic resilience during crises

**DOI:** 10.1002/ece3.8986

**Published:** 2022-06-16

**Authors:** Ignasi Arranz, Regina Nobre, Julien Cucherousset, Aline Reis de Carvalho, Amanda Cantarute‐Rodrigues, Pierre Favriou, Flavien Garcia, Marie Gimenez, Alexis Imbert, Valentin Marin, Ivan Paz‐Vinas

**Affiliations:** ^1^ Laboratoire Evolution et Diversité Biologique (EDB) UMR5174 Université Toulouse 3 Paul Sabatier CNRS IRD Toulouse France; ^2^ 42487 Programa de Pós‐Graduação em Ecologia de Ambientes Aquáticos Continentais Universidade Estadual de Maringá Maringá Brazil

**Keywords:** adaptability, crisis, remote working environment, research group, social restrictions, virtual lab

## Abstract

The COVID‐19 pandemic has heavily impacted academics’ professional and personal lives, forcing many research groups (labs) to shift from an academic system primarily based on in‐person work to an almost full‐time remote workforce during lockdowns. Labs are generally characterized by a strong lab culture that underpins all research and social activities of its members. Lab culture traditionally builds on the pillars of in‐person communication, knowledge sharing, and all social and professional activities that promote collaboration, team building, scientific productivity, and well‐being. Here, we use the experience of our research group facing the COVID‐19 pandemic to illustrate how proactively reinforcing lab culture and its positive outcomes have been essential to our lab when transitioning from an in‐person to a remote lab environment, and through its ongoing evolution toward a hybrid remote/in‐person model. We argue that the proactive promotion of lab culture in research groups can foster academic resilience during crises, helping research groups to maintain their capacity to conduct scientific activities while preserving a sustainable life/work balance and a healthy mental condition.

## Graphical abstract

The Covid‐19 pandemic crisis has forced many research groups to move from an academic system based on in‐person work and characterized by a strong lab culture to a fully remote workforce. We illustrate how reinforcing lab culture has been essential for our research group when transitioning from an in‐person to a remote lab. We argue that proactively promoting lab culture is essential for supporting academic resilience during crises and to help research groups maintain scientific activity and preserve a sustainable life/work balance.

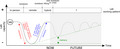

## 1 INTRODUCTION

**FIGURE 1:**
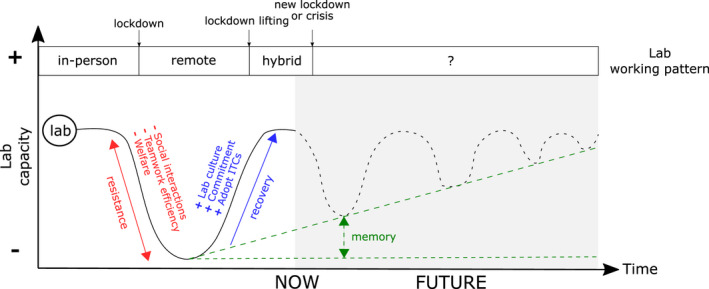
Hill and valley–like illustration of the effects of the COVID‐19 pandemic on the lab capacity and the importance of lab culture in promoting resilience. We transitioned from a lab working pattern mainly based on full‐time in‐person work to a full remote lab environment just before the lockdown of the second wave of the COVID‐19 pandemic in France. Our lab had to *resist* against the pervasive effects induced by the perturbation (i.e., the lockdown), which included drastic reductions in social and professional interactions, teamwork capacities, and lab members welfare. To *recover* to a sustainable and efficient level, we fostered our *resilience capacity* by proactively promoting lab culture, increasing our commitment to participate and develop team‐building activities, and adopting a set of complementary Information and Communications Technology Tools (ICTs). When the lockdown was lifted, we then transitioned to a hybrid lab that mixed in‐person and virtual interactions among all members. Our resilience capacity improved during the crisis, and we have improved our *memory* to better resist and recover from potential upcoming crises (shaded area)

As the COVID‐19 pandemic profoundly impacted professional and personal lives, society has faced challenges to adapt to new daily changes and to a new normality (Tesar, 2020). Like most working environments, academia has been deeply shaken for more than 2 years by this public health crisis that is still ongoing, and that has evolved through multiple COVID‐19 phases, ranging from lockdown periods (with limited social and in‐person interactions) to periods with relaxed social restrictions. Research laboratories (hereafter, *labs*) gather scientists at various career stages working under the umbrella of similar and/or complementary scientific topics. Labs are generally characterized by a solid *lab culture* that underpins all research activities (e.g., thesis defenses, fieldwork campaigns, and project meetings) and social interactions (e.g., coffee breaks, and retreats) of lab members. Lab culture integrates the culture of diversity, collaboration, and teamwork by bringing together complementary knowledge and skills from members who often have different expertise, experience, and scientific and cultural backgrounds (Genovesi, 2014; Maestre, 2019; Powell, 2020). The COVID‐19 pandemic suddenly affected labs’ capacities (i.e., the sum of all research activities carried out in a lab), and researchers had to adapt from an almost full‐time *remote* workforce during lockdown phases to partial‐to‐full in‐person work during phases with lifted social restrictions (Buchanan et al., 2021; Figure 1). The shift between different COVID‐19 phases led scientists to face drastic daily changes by adopting different working dynamics, potentially having negative impacts on lab's capacities due to reduced teamwork efficiency, interactions between lab members, and lab members’ welfare (Figure 1). Here, we posit that promoting lab culture, its positive outcomes, and the possibility to transition from physical to remote labs is essential for increasing the academic resilience during crises and efficiently maintaining research activity for scientists at different stages of their careers (Figure 1).

In Ecology, resilience is the capacity of a system to resist and recover from a perturbation (Holling, 1973). When a given system (e.g., a lab) is affected by a perturbation (e.g., the public health crisis induced by the COVID‐19 pandemic), it may (i) show more or less resistance to the changes induced by this perturbation, (ii) adopt strategies (e.g., actions promoting lab culture) to mitigate potential damages, and (iii) ultimately recover to sustainable levels of stability and efficiency (Figure 1; Folke et al., 2010; Rillig et al., 2020). Several important factors determining resilience when facing a crisis have been identified (Coulombe et al., 2020). Some of them involve socio‐ecological perspectives including trust in healthcare institutions (Ward, 2017), familiar support (Xiao et al., 2020), and institutional policies (Caplanova et al., 2021). In addition to these factors, the resilience of a lab when facing a crisis can also be developed by proactively promoting lab culture, notably during the most intense period of the crisis. Based on our experience during the COVID‐19 pandemic, we argue that succeeding to build a lab working environment with a strong lab culture can provide a buffer against the negative effects induced by crises (Figure 1).

Mitigating the effects of COVID‐19 phases on lab capacity was challenging for our lab – a group of freshwater ecologists at different career stages from undergraduate and graduate students to the principal investigator, including post‐doctorates, and a lab manager. Indeed, a large proportion of its members joined the lab remotely during the first two pandemic waves in France (March–October 2020) and, in some cases, the newcomers had to start their contracts from abroad or from different locations in France. Some members had to delay their relocation until the opening of the international borders and to combine their new work‐at‐home reality with childcare. It was just before the second lockdown (November 2020) when we discussed how to improve our ability to face a foreseeable upcoming lockdown, as no guidance and recommendation emerged from our institutions. In order to fill some institutional gaps, we collectively decided to develop a new working environment in our research group to face the upcoming phases of the COVID‐19 crisis by committing to proactively promoting lab culture (Figure 1), which was relatively lacking in our institutional environment.

Here, we present the actions we undertook during the COVID‐19 pandemic to promote lab culture when building a new remote lab environment, and to set a resilient working approach that fosters teamwork, scientific productivity, and well‐being during crisis times. We adopted strategies and we built a collective *memory* that could be recalled during future crises to better resist and recover from perturbations (Figure 1). Our experience may help to face future crises and develop a more flexible and pronounced lab culture in academia during its transition toward a hybrid remote/in‐person model (Srivastava et al., 2021).

## 2 ACTIONS TO PROMOTE LAB CULTURE AS ACADEMIC RESILIENCE DURING CRISES

Setting up a remote lab environment (i.e., a virtual space to share, discuss and conduct research tasks) was the most efficient tool for promoting a strong lab culture, as it allowed us to exchange knowledge, plan inter‐lab meetings, and foster collaborations, while simultaneously providing an environment for informal discussions and networking. The adoption by all lab members of Information and Communications Technologies (ICTs) was key for setting up our remote lab environment (Figure 1), in the same way as they successfully contributed to the development of online education (Chakraborty et al., 2021). For example, business communication platforms and digital file‐sharing tools greatly facilitated resource sharing and accessibility, communication, and social relationships among the lab members (Figure 1). Creating a flexible, but committed schedule with regular virtual lab meetings, coffee breaks for informal discussions, and project‐oriented meetings helped us promoting lab culture while providing a space to warmly welcome and integrate newcomers despite social distancing (Powell, 2020). From our experience, sticking to weekly virtual lab meetings was key to build a space where we updated our personal and professional goals, shared our achievements and difficulties, discussed research, and gathered inputs from other lab members. Moreover, the commitment to regular project‐oriented meetings allowed us to overcome the difficulties and challenges of online supervision, helping to satisfy both supervisor and supervised parties’ expectations. We also were committed to creating forum channels dedicated to free subjects (*No Topic is Banned* was the motto) such as lightning trainings, programming tutorials, or even broad discussions on movies and books. However, not all our initiatives to promote lab culture were successful. For instance, we created virtual coffee rooms where lab members could meet and chat informally at specific hours, but this initiative was poorly adopted by lab members. This was likely because we did not yet know each other sufficiently, or because we tried to formalize a typically informal activity. This initiative could have worked if we had dedicated more time for one‐on‐one encounters or speed meetings at the beginning of the crisis, or by increasing the frequency of online meetings (e.g., two or three meetings per week, with a series of 20‐ to 30‐minute informal talks), with some of these meetings oriented toward engaging team building (Stürmer et al., 2006). Other informal (virtual) lab experiences that could have been implemented range from reading poetry or sharing home‐made recipes (Cénat et al., 2020) to transforming all meetings such as data clubs, journal clubs, and even pub meetings to online formats (Chacón‐Labella et al., 2021; Rillig et al., 2020).

Promoting lab culture in remote lab environments can also increase academic resilience by facilitating collaboration and discussion with international researchers (e.g., through cross‐group meetings or initiatives like coordinated readings or journal clubs; Lee & Haupt, 2021; Duan & Xia, 2021; or helping to broaden social and professional networks; Lortie, 2020; Trogisch et al., 2020). Remote lab meetings facilitate the involvement of overseas guests who can present their research, fuel scientific discussions, provide a different perspective on the way of doing research, or provide input on more general topics (e.g., how they build their career paths). From our experience, we invited and benefitted from the talks of former lab members and from researchers from different countries, while some of the members of our lab gave remote talks or seminars in other labs. As the COVID‐19 public health crisis has shown us how the future can be uncertain, we believe that the ability of research groups to be flexible and their capacity to shift from in‐person to remote lab environments will help them mitigate the negative impacts of potential crises on research activities and lab members. An example to acquire this flexibility might be to maintain or develop the use of remote lab environments during no‐crisis periods, even when in‐person lab activities are the norm (e.g., by maintaining a background use of business communication platforms and other ICTs).

## 3 LAB CULTURE TO IMPROVE HUMAN HEALTH AND WELFARE

An old proverb says that *It is better to light a candle than to curse the darkness*. Promoting lab culture not only can help improve academic resilience during a crisis but it can also become a solution to cope with two important psychological issues in academia (González‐Sanguino et al., 2021; Talevi et al., 2020). First, it may help to prevent emotional exhaustion, depersonalization, and burnout syndrome in lab members, which may negatively impact individuals’ health and professional trajectories (Gewin, 2021; Maslach & Jackson, 1981; Maslach & Leiter, 2016). Burnout syndromes and drop‐outs might be exacerbated when working remotely in isolation (Abbott, 2021; Brooks et al., 2020). However, even during periods of social restrictions such as COVID‐19 lockdown periods, maintaining a strong lab culture in remote lab environments can provide a healthy working space that may connect researchers worldwide to share their own experiences while managing the time and interactions among people in their labs. Second, integrating lab culture into remote lab environments can help mitigate anxiety and depression, two common mental conditions that have also recently risen in the competitive world of academia, and that consistently affect all researchers at different career stages (Evans et al., 2018; Woolston, 2018). Previous works have observed that people who had the closest relationships felt a sense of social belonging, and suffered fewer symptoms of depression (Steger & Kashdan, 2009; Waldinger & Schulz, 2010). Thus, building a stronger lab culture by stimulating within‐group members’ relationships and social belonging and by fostering extra‐group relationships and collaborations can help researchers to prevent, detect earlier, and cope with mental health problems, even in cases where social distance prevails. As we are still shifting between periods of in‐person and remote work, and because remote working will be more common in the next years, developing social interactions by offering flexible tools or environments capable of adapting toward a more versatile, healthy, and resilient academic system will have positive outcomes in the individual's welfare.

## 4 CONCLUSIONS

Rising academic resilience by proactively developing lab culture was essential to our lab for overcoming the COVID‐19 pandemic, and will likely be important in the future to cope with other potential crises. Our engagement in regular virtual meetings, the adoption of ICT tools, the overall commitment of all lab members to participate in team‐building initiatives, and the involvement of invited guests were efficient strategies to build a remote lab environment with a marked lab culture that helped us to increase our academic resilience. Other crises such as climatic disasters or territorial disputes can also emerge and affect professional and personal lives in the future. Because crises can disrupt working environments at any time, we need to constantly adapt flexible thinking strategies to help preserve and recover lab capacities when facing crises through building a collective memory. Proactive attitudes and constant development and testing of new strategies to build flexible, healthy, and efficient lab working environments will therefore positively affect the resilience of research groups, increasing their capacity to mitigate potential crises’ consequences on lab members’ well‐being and on lab capacities.

## AUTHOR CONTRIBUTIONS

**Ignasi Arranz Urgell:** Conceptualization (lead); Writing – original draft (equal); Writing – review & editing (equal). **Regina Nobre:** Writing – original draft (equal); Writing – review & editing (equal). **Julien Cucherousset:** Writing – review & editing (equal). **Aline Carvalho:** Writing – review & editing (equal). **Amanda Cantarute‐Rodrigues:** Writing – review & editing (equal). **Pierre Favriou:** Writing – review & editing (equal). **Flavien Garcia:** Writing – review & editing (equal). **Marie Gimenez:** Writing – review & editing (equal). **Alexis Imbert:** Writing – review & editing (equal). **Valentin Marin:** Writing – review & editing (equal). **Ivan Paz‐Vinas:** Writing – original draft (equal); Writing – review & editing (equal).

## CONFLICT OF INTEREST

The authors declare no competing interests.

## Data Availability

No datasets were analyzed or generated during the current study.
